# Descriptive transcriptomic profiling differentiates oral leukoplakia from proliferative verrucous leukoplakia and reveals distinct molecular signatures

**DOI:** 10.4317/medoral.27658

**Published:** 2025-10-17

**Authors:** Mario Pérez-Sayáns, Fábio França Vieira-e-Silva, Ceres Fernández-Rozadilla, Ángel Carracedo, Silvia Carlés-González, Alejandro Ismael Lorenzo-Pouso, Alba Pérez-Jardón, Pilar Gándara-Vila, Abel García-García, José Manuel Suárez-Peñaranda, Andrés Blanco-Carrión, Cintia Micaela Chamorro-Petronacci

**Affiliations:** 1 Oral Medicine, Oral Surgery and Implantology Unit (MedOralRes), Faculty of Medicine and Dentistry, University of Santiago de Compostela, San Francisco Street, s/n, 15782 Santiago de Compostela, Spain; 2 Health Research Institute of Santiago de Compostela (FIDIS), ORALRES Group, Santiago de Compostela University Clinical Hospital, University of Santiago de Compostela, Choupana Street, s/n, 15706 Santiago de Compostela, Spain; 3 Materials Institute of Santiago de Compostela (iMATUS), Mestre Mateo Avenue, 25, 15782 Santiago de Compostela, Spain; 4 Health Research Institute of Santiago de Compostela (FIDIS), Genomic Medicine Group, s/n, Choupana Street, 15706 Santiago de Compostela, Spain

## Abstract

**Background:**

Oral leukoplakia and proliferative verrucous leukoplakia represent oral potentially malignant disorders. Oral leukoplakia typically presents as solitary lesions, while proliferative verrucous leukoplakia manifests as multifocal lesions with higher malignant potential. This study aimed to investigate the genetic heterogeneity between these disorders through differential gene expression, genetic variants, and microRNA profiling to identify potential biomarkers for diagnosis and prognosis.

**Material and Methods:**

Biopsies and peripheral blood samples were obtained from 20 patients. Subsequently, RNA extraction, RNA-Seq libraries preparation, and bioinformatic analyses were conducted to ascertain differential gene expression, genetic variants, and microRNA expression.

**Results:**

In mRNA analysis, overexpressed genes in proliferative verrucous leukoplakia are primarily associated with inflammation and immune regulation, while underexpressed genes relate to skin barrier maintenance. Pathway analysis reveals underexpressed genes related to impaired keratinization in proliferative verrucous leukoplakia and with keratin envelope formation in oral leukoplakia, while overexpressed genes are linked to synaptic processes and protein-protein interactions. Somatic mutation drivers in proliferative verrucous leukoplakia include variants in NRXN3, SRGAP2B, INIP, MYO18A, and ATF7IP genes. Regarding variant analysis, two variants in the Syndecan 3 (SDC3) gene identified in proliferative verrucous leukoplakia have demonstrated enormous value and indicate an important biomarker for a differential diagnosis and to predict prognosis. Proliferative verrucous leukoplakia shows in miRNA analysis MIR1246 and MIR767 overexpression, with MIR135B being the most underexpressed.

**Conclusions:**

Our findings emphasize the intricate transcriptomic profiles in oral leukoplakia and proliferative verrucous leukoplakia development, laying the groundwork for future studies to enhance clinical management and patient outcomes in oral oncology. Syndecan 3 gene polymorphisms may represent a key point in proliferative verrucous leukoplakia differential diagnosis and prognostic prediction.

## Introduction

Oral leukoplakia (OL) and proliferative verrucous leukoplakia (PVL) are oral potentially malignant disorders (OPMD), representing a significant concern in the oral pathology field (1). PVL corresponds to the OPMD with the greatest potential for malignancy (66-70%) compa-red to OL (7.9-11.7%). These lesions share remarkable similarities in clinical presentation and histopathology, highlighting challenges in differentiating between them and underscoring the importance of searching for novel biomarkers (1). This study employed RNA-Seq to analyze the differential gene expression profiles and genetic variations in OL and PVL to elucidate the molecular mechanisms driving their origin and clinical behavior.

## Material and Methods

Patients

Samples were obtained from 20 patients diagnosed clinically and histopathologically with OL (n=15) or PVL (n=5); From each patient, one fresh biopsy and one peripheral blood sample were collected. Patients were referred to the Oral Medicine Unit of the Faculty of Medicine and Dentistry at the University of Santiago de Compostela and had at least 24 months of follow-up.

Incisional biopsies of 5×5mm from different lesions were taken using a scalpel and stored in Allprotect Tissue Reagent (Qiagen, Hilden, Germany) at -80ºC until analysis. Simultaneously, 10 mL of peripheral venous blood was obtained from each patient according to the standard protocol.

In the PVL group, 20% of patients were male and 80% female, while in the OL group, 64.3% were male and 35.7% female. The mean age at diagnosis was 67.9 years in PVL (range 52.5-82.8) and 62.68 years in OL (range 26.5-85.2). All PVL cases were non-homogeneous, whereas in OL, 64.3% were homogeneous and 35.7% non-homogeneous. Lesions in PVL occurred on the tongue (40%), palate (20%), and gum (40%). In OL, the tongue was affected in 50%, buccal mucosa in 35.7%, palate in 7.1%, and gum in 7.1%. Dysplasia in PVL was absent in 60% and low-grade in 40%. In OL, dysplasia was absent in 50%, low-grade in 35.7%, high-grade in 7.1%, and inconclusive in 7.1%. Clinical evolution in PVL showed cure in 80% and stability in 20%. In OL, 28.6% were cured, 42.9% stable, 21.4% showed malignization, and 7.1% had recurrence. All PVL patients were alive at last follow-up, while in OL, 92.9% were alive and 7.1% had died.

Ethical Aspects

Before collecting samples, each patient received the study's information sheet and signed an informed consent form, agreeing to participate in the study. They were treated in accordance with the Declaration of Helsinki and its later amendments. This study was ap-proved by the clinical research and drug ethics committee of Galicia (Ref. 2018/503).

RNA Extraction

The RNA extraction was performed using the mirVana miRNA Isolation Kit (Ambion, Austin, TX, USA), and RNA was treated with DNAse I, using the DNA-free DNA Removal Kit (Invitrogen, Thermo Fisher Scientific, Waltham, MA, USA), following the manufacturer's protocols. The RNA quality was analysed by the RNA integrity number (RIN) values with an Agilent 2100 Bioanalyzer system.

RNA-Sequencing (RNA-Seq)

TruSeq Stranded Total RNA libraries were constructed by Macrogen Inc. (Macrogen Europe, Amsterdam, The Netherlands) to obtain Illumina paired-end reads (151 bp read length).

RNA-Seq quality control was performed using Fastqc for each FASTQ file and Multiqc to integrate sample results (http://www.bioinformatics.babraham.ac.uk/projects/fastqc/). Per Sequence Quality Scores, Sequence Duplication Levels, and Overrepresented Sequences were considered. Reads were aligned to the human genome (GRCh38) using STAR (2), and gene counts (GC) were estimated using featureCounts (3). Differential expression gene (DGE) analyses were conducted using R Software (R Core Team 2021, Vienna, Austria). Count values were imported and processed using edgeR (4). Expression values were normalized using the trimmed mean of M values (TMM) method, and lowly-expressed genes were filtered out.

Genetic Variant Evaluation

Mutations were described by mRNA variant, associated gene, cDNA position, and nucleotide mutation. Scale-Invariant Feature Transform (SIFT) and Polymorphism Phenotyping (PolyPhen) were performed as bioinformatic tools for functional impact predictive assessment.

DGE Analysis

DGE was identified using linear models (Limma-Voom) (5), and P values were adjusted for multiple comparisons by applying the Benjamini-Hochberg correction. Heatmaps and volcano plots were generated using the heatmap3 (6), EnhancedVolcano (https://github.com/kevinblighe/EnhancedVolcano), and Glimma (7) packages.

Pathway Analyses

Gene Set Enrichment Analysis (GSEA) techniques, specifically Gene Ontology Biological Process (GOBP) and REACTOME (molecular pathways) were used to indicate major functional enrichments for different over- and underexpressed genes (OE and UE).

Gene Functional Annotation

Gene functional annotation was performed using clusterProfiler (8). Receiver Operating Characteristic (ROC) analyses were conducted using verification (https://cran.r-project.org/web/packages/verification/index.html).

DNA/Somatic Variant Analyses

Variant calling was performed using Samtools, and Bcftools, as previously reported (9). Variants in positions with coverage below 10 for either diseased or matched control samples were discarded. Disease-associated variants were established by comparing the variants of each diseased sample with its matched healthy control. Odds ratios (OR) were evaluated using Fisher's exact tests, and P values were adjusted for multiple comparisons by applying the Benjamini-Hochberg correction. The Ensemble Variant Effect Predictor (VEP) was used for variant annotation (10).

## Results

The quality control and normalization are demonstrated in Supplementary Figure 1-4 (http://www.medicina.oral.com/carpeta/suppl1_27658).

Differential Expression mRNA

Differential Gene Expression (DGE)

**Figure 1:**
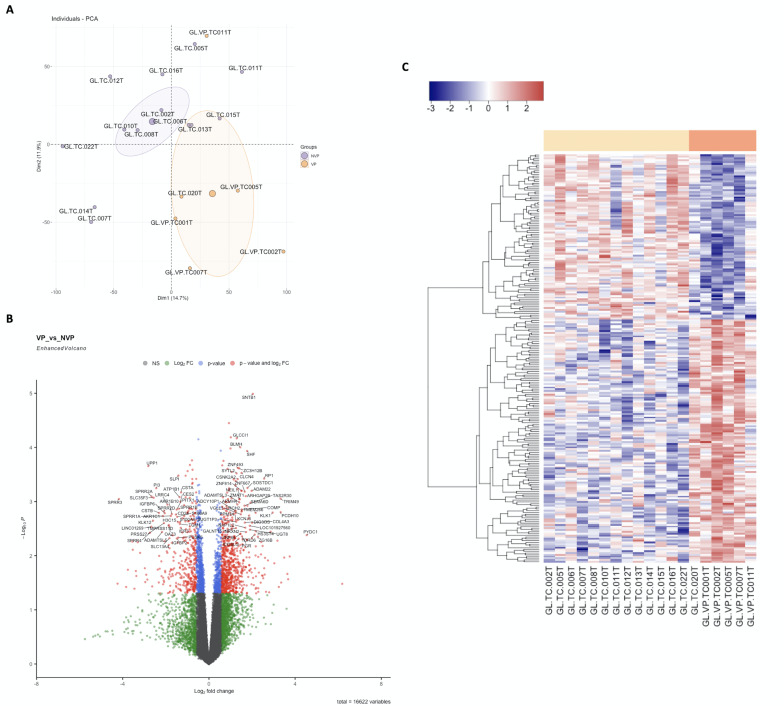
Differential Gene Expression (DGE) of mRNA in Oral Leukoplakia (OL) Versus Proliferative Verrucous Leukoplakia (PVL). (A) Principal Component Analysis (PCA) reflects a clear separation between biological groups, although intra-group dispersion is notable; (B) Volcano Plot; (C) and heatmap showing DGE represented in red for overexpressed genes and in blue for underexpressed genes, demonstrating clear clustering among different samples.

Principal Component Analysis (PCA) reflects a clear separation between biological groups (Figure 1A). Differential expression of the PVL group versus the OL group is calculated, with 138 OE genes and 93 UE genes. Genes showing differential expression are represented in red (OE) and blue (UE) in a volcano plot (Figure 1B), demonstrating clear clustering among different samples and in a heatmap (Figure 1C). For OE genes, the main altered biological pathway and function would be Inflammation and Immunity regulation, represented by PYDC1, TRIM49, and KLK1 genes. For UE genes, the skin barrier formation and maintenance would be affected, with SPRR2D, SPRR1A, SPRR2A, SPRR4, and SPRR3 genes, belonging to the proline-rich proteins family, being UE. No statistically significant associa-tion has been found with dysplasia degree. Regarding clinical evolution, two genes with differential expression have been found. Comparing patients who progressed to malignancy with those who remained stable, the C1orf226 gene (logFC=2.473, p=6.39E-06) was significantly OE, and the CYB5A gene (logFC=-3.209, p=8.732E-06) was UE.

1

Pathway analyses

GSEA techniques indicate the main functional enrichments for different genes. For UE genes, the main biological processes involved were keratinization, peptide cross-linking, keratinocyte differentiation, epidermal cell differentiation, and epidermis development, suggesting the impaired keratinization role in PVL lesions. Regarding molecular pathways, the cornified envelope and keratinization formation are confirmed as the primary pathways involved in UE genes. For OE genes, synaptic processes, and protein-protein interaction pathways are related, with SUMOylation being one of the most prevalent (ratio 0.0625) (Supplementary Figure 5 - http://www.medicina.oral.com/carpeta/suppl1_27658).

Somatic mutation drivers identification for PVL

In parallel to gene expression analyses, an RNA-based variant was performed to elucidate somatic alterations that are driving OL and PVL formation, as well as those that can differentiate both lesion types. Regarding PVL, our findings demonstrate some variants with frequent occurrence, considering those with a frequency higher than 0.6. The results demonstrate that the variants rs31437 in the NRXN3 gene (Frequency: 0.66, OR for NVP=0; p=0.0003); rs1478770644 in the SRGAP2B gene (Frequency: 0.50); rs557699893 in the INIP gene (Frequency: 0.50); rs8076604 in the MYO18A gene (Frequency: 0.50) and rs2231909 in the ATF7IP gene (Frequency: 0.50) are exclusive to PVL. ROC analysis identi-fied three highly predictive genes: ENSG00000172164 (SNTB1), AUC=1, adjusted p-value=0.002137697, ENSG00000182700 (IGIP), AUC=1, adjusted p-value=0.002137697, and ENSG00000116396 (KCNC4), AUC=1, adjusted p-value=0.002137697. The ROC curve analysis comparing malignancy and non-malignancy groups has no significant results for the studied genes.

Different Variant Analysis (DVA)

**Figure 2:**
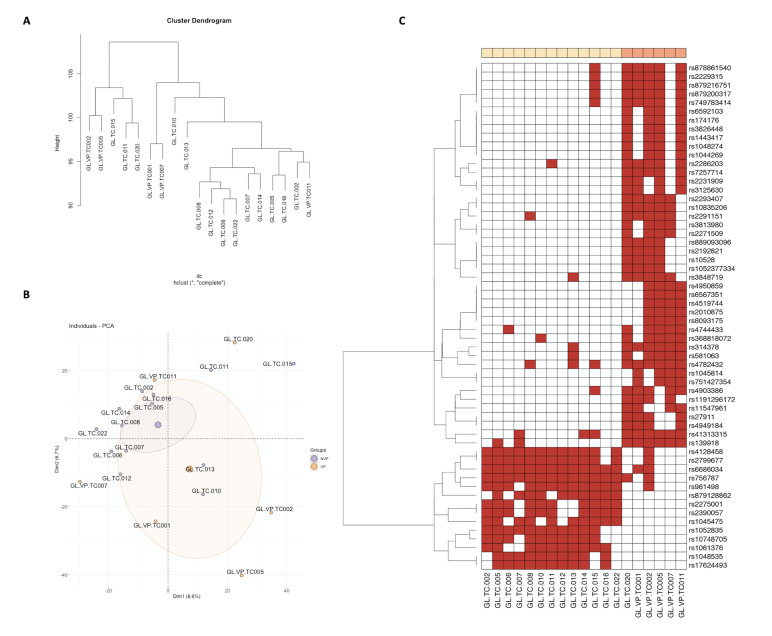
Different Variant Analysis (DVA). (A) Hierarchical clustering (dendrogram) reveals distinct groupings based on variants. Two main clusters demonstrate, one on the left, formed by samples with similar variants and consisting of OL that did not malign from diagnosis throughout the follow-up, and another on the right with all the other OLs including the 3 that evolved into a malignant tumor, and the one that presented a previous in situ carcinoma before the study, with a subcluster that groups two PVLs, GLVP.01 and GLVP.07, with a similar height to the other PVLs. (B) Principal Component Analysis (PCA) for DVA demonstrates a greater dispersion of samples in the PVL group, while a very high percent-age of OLs are grouped in the same area. (C) Heatmap for DVA demonstrates many common or nearly specific genetic variants of PVL compared to OL number.

The DVA is carried out similarly to DGE, revealing differentially enriched variants between the PVL and OL groups. Hierarchical clustering reveals distinct groupings based on variants (Figure 2A). We observe two clusters, one on the left, formed by samples GLVP.02/GLVP.05/GL.015/GL.011/GL.020, with similar variants and consisting of OL that did not malign from diagnosis throughout the follow-up. On the right would be another cluster with all the other OLs, including the 3 that evolved into a malignant tumor and the one that presented a previous in situ carcinoma before the study (GLVP.01). Within this right cluster, there is a small subcluster that groups two PVLs, GLVP.01 and GLVP.07, with similar height to the other PVLs.

2

In the PCA for DVA, there is a greater sample dispersion in the PVL group, while a very high percentage of OLs are grouped in a restricted area. Note the distance to the subcluster of samples GL.015, GL.011, and GL.020, which had a favorable clinical course (Figure 2B).

In the DVA heatmap, there is a large number of common or nearly specific genetic PVL variants, among the most significant are rs314378, rs139918, rs10835206, rs2293407, rs7257714, while for the OL group, there are smaller variants number but grouping almost all non-verrucous leukoplakias. GL.020 presents a variant profile most similar to PVLs, which can be clinically correlated as the patient has 3 lesions in different locations (Figure 2C).

Regarding variant annotation among the variants showing differential enrichment, In the PVL group, a missense variant in SDC3 (rs4949184, 1:30874552) was identified. The A allele resulted in an aspartic acid to tyrosine substitution (D/Y) with moderate predicted impact. Two transcripts were affected (protein positions 245 and 303), both classified as deleterious by SIFT (score 0) and predicted as probably damaging (PolyPhen 0.962) or possibly damaging (PolyPhen 0.888). In the OL group, a missense variant in FBLN2 (rs1061376, 3:13637835) led to an aspartic acid to glutamic acid substitution (D/E). This variant was detected in three transcript positions (protein positions 1157 and 1204), consistently predicted as deleterious by SIFT (0.01-0.02) and probably damaging by PolyPhen (0.94-0.998). Additionally, a missense variant in LAD1 (rs2799677, 1:201386815) was observed, corresponding to a lysine to asparagine substitution (K/N). It was found in three transcript positions (protein positions 182 and 196) and classified as deleterious by SIFT (score 0, one low-confidence) and possibly damaging by PolyPhen (0.582-0.736).

miRNA Analysis

Differential miRNA expression

**Figure 3:**
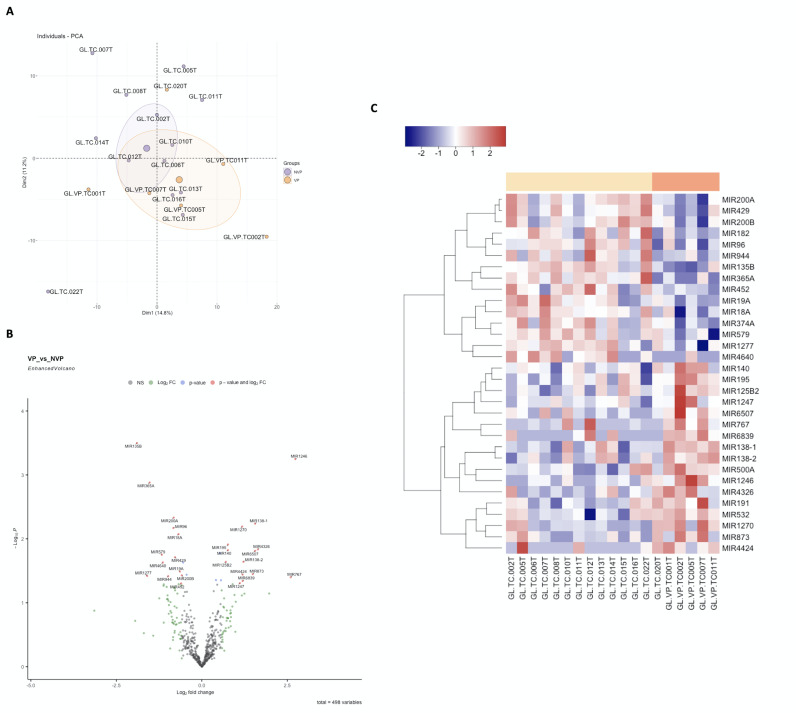
Differential Gene Expression (DGE) of miRNA in Oral Leukoplakia (OL) Versus Proliferative Verrucous Leukoplakia (PVL). (A) Principal Component Analysis (PCA) reflects high intra-group dispersion, especially in the OL group, although less in the PVL group; (B) Volcano Plot; (C) and heatmap showing DGE represented in red for overexpressed genes and in blue for underexpressed genes.

PCA analysis (Figure 3A) reflects high intra-group dispersion, especially in the OL group. Genes showing differential expression are indicated in red (OE) and blue (UE). These genes are represented in a volcano plot heatmap (Figure 3B) and a heatmap (Figure 3C). It is noteworthy MIR1246 and MIR767 OE. No UE miRNA with an FC &lt;2 is described, with MIR135B being the last on the list. Regarding follow-up, some OE miRNAs are described in cases that undergo malignization: MI767 (FC=3.56; p=0.0358), MIR208B (FC=3.29; p=0.033), MIR208A (FC=2.54; p=0.0042), MIR518C (FC=2.07); p=0.0253), MIR4798 (FC=2.06; p=0.0150). One UE miRNA with FC &lt;2 is described, MIR365B (FC=-2.21; p=0.0084).

3

Functional Annotation

Functional enrichments for different UE and OE miRNAs are indicated in Supplementary Figure 6 (http://www.medicina.oral.com/carpeta/suppl1_27658).

For UE genes, the main biological process involved was the translation regulation, mainly blocking protein production. For OE genes, muscle apoptosis processes and negative vascular endothelial growth factor regulation are primarily related.

## Discussion

Our findings on mRNA DGE reveal that OE genes in PVL were mainly related to inflammation and immune regulation, in contrast, UE genes were associated with forming and maintaining the skin barrier. Llorens et al. also found significant upregulation of genes associated with immune processes, inflammation, and defense responses in PVL, emphasizing the role of immune modulation through binding proteins and receptors (11). A plausible hypothesis to explain the immune response in PVL patients may be related to microbiological infection (12).

Regarding pathway analyses, UE genes suggest impaired keratinization's role in PVL lesions, while in OL, processes such as keratin envelope formation were highlighted. For OE genes, synaptic processes and protein-protein interaction pathways are related. Farah et al., when performing pathway analysis, presented similar results, demonstrating that the pathways related to epithelial-mesenchymal transition (EMT) were significantly altered (13). Kyrodimou et al., in their study focusing on the differences in Desmoglein-3/-catenin and E-cadherin/ß-catenin expression emphasize an association with the dysplasia degree and the differentiation stage (14).

In our study several specific PVL variants somatic mutation drivers were found, the main ones being: rs31437 in NRXN3, a gene linked to neuropsychiatric disorders (15); rs1478770644 in SRGAP2B, associated with synaptic density and maturation, impacting neurological development (16); rs557699893 in INIP, crucial for genome stability and disease susceptibility (17); rs8076604 in MYO18A, involved in maintaining muscle cell stability (18); rs2231909 in ATF7IP, related to immune functions and cancer (19).

Through ROC analysis in identifying somatic mutation drivers for PVL, three genes with high predictive capacity were identified: SNTB1, IGIP, and KCNC4. Zhang et al. demonstrated that SNTB1 positively regulates the expression of YAP1, affecting tumor growth and metastasis in colorectal cancer (20). Austin et al. dissected IGIP functions and described that this gene regulates IgA by acting as a switch or differentiation factor (21). In head and neck squamous cell carcinoma (HNSCC), it is reported that there is an increase of KCNC4 mRNA levels in tumors compared to the corresponding normal epithelia (22).

In our analysis related to DVA analysis, two variants in the SDC3 gene identified in patients with PVL are of particular interest. Syndecans are a family of four transmembrane heparan sulfate proteoglycans, including syndecan -1, -2, -3 and -4. SDC3 is mainly expressed in brain and nervous tissue, with a key role in development, cell adhesion, and migration (23). Although some Syndecan family members are already widely debated in the literature, SDC3 is a gene that needs more information, with no association with OL or PVL previously described. In contrast, Syndecan 1 has been associated with oral dysplastic lesions (24), OSCC (25), and oral verrucous carcinoma (26).

Hudák et al. identified SDC3 expression of monocytes as a novel biomarker for early Alzheimer's disease detection (27). Brito et al. described the power of SDC3 in enhancing WNT signaling in osteoblasts, making SDC3 an attractive target for novel bone anabolic drug development (28). Kehoe et al. demonstrated the role of SDC3 in inflammation, acting as a pro-inflammatory agent in the endothelial presentation of chemokines and the leukocytes recruitment and in cartilage damage in a rheumatoid arthritis model, unlike in the skin and cremaster, where its action is anti-inflammatory (29).

In cancer research, Prieto-Hernández et al. demonstrated that SDC3 expression is upregulated in several types of cancer, especially in hypoxic solid tumors (30). Hillemeyer et al. suggest that SDC3 upregulation promotes ovarian cancer pathogenesis by modulating stemness-associated pathways (31). Yamada et al. showed that the antitumor miR-144-5p/oncogenic SDC3 axis was deeply involved in renal cancer pathogenesis (32).

In the OL group, we found variants in two genes, FBLN2 and LAD1. FBLN2 is involved in carcinogenesis through interaction with other extracellular matrices (ECM) proteins, including integrins and syndecans, with pathways related to the elastic fibers formation and ECM organization, being related to diseases such as Osteochondritis Dissecans and Cutis Laxa (33). LAD1 is a gene classified as collagen-anchored filaments in the basement membrane of mammalian epidermal cells that contributes to the stability of epithelial layers associated with the underlying mesenchyme, being correlated with diseases such as Epidermolysis Bullosa Acquisita and Iga Linear Disease (34). Farah et al. explored the biological implications of the DGE between OL with and without dysplasia, describing that the negatively regulated genes were significantly enriched considering the biological processes related to collagen fibrils organization and ECM (13).

In our miRNA analysis, the PVL group DGE compared to the non-PVL group highlighted that MIR1246 and MIR767 are OE, with MIR135B being the most UE. miR-1246 has been reported to regulate several pathways, and miR-767 is highly expressed in senescent vascular endothelial cells and their exosomes (35). Choi et al. demonstrated that blocking miR135b improves the microenvironment and basal cell proliferation in psoriasis, demonstrating a central role for miR135b in epidermal keratinocytes (36).

When analyzing the malignancy process, our results demonstrate that MI767, MIR208B, MIR208A, MIR518C, and MIR4798 are OE, while MIR365B is UE. In contrast, Zhu et al. identified a series of other 8 miRNAs related to OL to OSCC evolution (37). Chen et al. also demonstrated different results with miR-129-5p, miR-296-5p, and miR-450b-5p commonly regulated between OL and OL-OSCC (38).

Our research involves identifying RNA variants from mucosal and blood samples instead of DNA. Consequently, the primary limitation is the potential presence of variants in the mucosa that may not be found in other tissues due to the differing expression profiles between tissues rather than mutations necessarily associated with the pathology. Ideally, variants should be identified from DNA or, failing that, use a germline control from the same tissue if RNA (healthy mucosa rather than blood) is desired. However, in OPMD patients, the oral mucosa behaves like a precancerous field. Therefore, the use of healthy mucosa as a control is also not ideal. Despite some limitations, our study offers important multi-omic insights into distinguishing OL from PVL beyond clinical characteristics.

## Conclusions

Differential mRNA expression analysis revealed distinct OE and UE genes between PVL and OL, with key pathways involving inflammation, immune response, and epithelial barrier maintenance. Enrichment analyses emphasized keratinization-related processes in PVL and cornified envelope formation in OL. Somatic mutation analysis uncovered PVL-specific variants, including in the ATF7IP and MYO18A genes, suggesting their relevance to pathogenesis and diagnostic potential. Notably, SNTB1, IGIP, and KCNC4 showed high predictive value for PVL. Variant analysis also identified two significant SDC3 variants in PVL, supporting their role as potential diagnostic biomarkers. miRNA profiling revealed differentially expressed miRNAs, some associated with malignant transformation. Functional annotation highlighted biological processes such as muscle apoptosis and translational repression. Altogether, these findings offer molecular insights into PVL and OL, laying the groundwork for future diagnostic and therapeutic strategies.

## Data Availability

Data is available upon request from the authors.
